# Psychological Stress and Male Infertility: Oxidative Stress as the Common Downstream Pathway

**DOI:** 10.3390/biomedicines14020259

**Published:** 2026-01-23

**Authors:** Aris Kaltsas, Stamatis Papaharitou, Fotios Dimitriadis, Michael Chrisofos, Nikolaos Sofikitis

**Affiliations:** 1 Third Department of Urology, Attikon University Hospital, School of Medicine, National and Kapodistrian University of Athens, 12462 Athens, Greece; ares-kaltsas@hotmail.com (A.K.); mchrysof@med.uoa.gr (M.C.); 2 Andrology Laboratory and Sperm Bank, 54622 Thessaloniki, Greece; sdrcauth@gmail.com; 3 Department of Urology, Faculty of Medicine, School of Health Sciences, Aristotle University of Thessaloniki, 54124 Thessaloniki, Greece; helabio@yahoo.gr; 4 Laboratory of Spermatology, Department of Urology, Faculty of Medicine, School of Health Sciences, University of Ioannina, 45110 Ioannina, Greece

**Keywords:** psychological stress, oxidative stress, male infertility, reactive oxygen species, sperm DNA damage, inflammation, lifestyle factors

## Abstract

Psychological stress is increasingly investigated as a potentially modifiable factor in male infertility, in part through oxidative stress. This narrative review synthesizes mechanistic and translational evidence linking stress-related neuroendocrine activation and coping behaviors with redox imbalance in the male reproductive tract. Chronic activation of the hypothalamic–pituitary–adrenal axis and sympathetic outflow elevates glucocorticoids and catecholamines. In controlled animal stress paradigms, this is accompanied by suppression of the hypothalamic–pituitary–gonadal axis and by immune and metabolic changes that favor reactive oxygen species generation. The resulting oxidative stress may reduce Leydig cell steroidogenesis, impair testicular and epididymal function, and induce lipid peroxidation, mitochondrial dysfunction, and sperm DNA fragmentation. In such models, these lesions, together with apoptosis of germ and supporting cells, are associated with lower sperm concentration, reduced motility, compromised viability, and diminished fertilizing potential. Overall, preclinical animal studies using defined stress paradigms provide experimental evidence consistent with causal effects of stress on oxidative injury and reproductive impairment in preclinical settings. Human studies linking perceived stress, anxiety/depression, and disturbed sleep to adverse semen parameters and oxidative biomarkers are summarized. However, the human evidence is predominantly associative, and the available studies are cross sectional and remain vulnerable to residual confounding and reverse causality. Potential effect modifiers, including smoking, alcohol use, and circadian disruption, are also discussed as contributors to heterogeneity across clinical studies. Standardized assessment of stress biology and redox status, longitudinal designs aligned with spermatogenic timing, and well-powered intervention trials are needed to define dose–response relationships and support individualized prevention and care.

## 1. Introduction

Infertility is increasingly recognized as a major and still under-addressed public health challenge, with substantial consequences for health, relationships, and quality of life. The World Health Organization (WHO) has recently estimated that approximately one in six people of reproductive age experience infertility during their lifetime and has highlighted the profound financial and psychosocial burden borne by individuals and couples seeking care [1]. Within this broader context, the male partner contributes meaningfully to the burden of involuntary childlessness. Contemporary European guidance indicates that a male-infertility-associated factor is identified in about half of involuntarily childless couples, reinforcing the importance of systematic male evaluation and of prioritizing modifiable exposures that may influence male reproductive potential [2].

Beyond biomedical diagnosis, infertility and fertility treatment trajectories are strongly intertwined with psychological well-being. A growing body of literature emphasises that men frequently experience distress that is clinically relevant yet often under-detected in routine fertility care. In a recent systematic analysis, case–control studies reported that men with male factor infertility exhibit more symptoms of depression, anxiety and general psychological distress than fertile controls, supporting the need for fertility services to view men as individuals with distinct psychosocial support needs rather than solely as partners in a couple-based pathway [3]. Consistent with this, recent quantitative synthesis suggests that anxiety symptoms are common in infertile men, with a pooled prevalence of approximately 21% across studies [4]. Aligning with these data, the WHO has explicitly underscored that infertility can impair mental and psychosocial well-being and has called for ongoing access to psychosocial support as a component of people-centred infertility care [1].

In parallel with the expanding attention to psychosocial dimensions of infertility, oxidative stress, defined here as a sustained redox imbalance in which oxidant activity exceeds enzymatic and nonenzymatic antioxidant defenses, has consolidated its role as a key construct in contemporary andrology [5]. Reactive oxygen species are short lived oxidant intermediates that can arise from sperm, leukocytes, and reproductive tissues, whereas oxidative damage refers to downstream lesions such as lipid peroxidation, protein oxidation, and oxidative DNA base modifications [5]. This framework has motivated interest in scalable approaches to characterize the seminal oxidative milieu. In a recent systematic review and meta-analysis, seminal oxidation–reduction potential (ORP) was lower in fertile men than in men with infertility and showed consistent associations with multiple semen quality parameters, supporting ORP as a promising integrated marker of overall redox state and oxidative stress, rather than a direct measurement of ROS concentration [6]. 

These converging lines of evidence frame an important translational question: how psychological stress and stress-related coping behaviors intersect with oxidative processes to shape male reproductive outcomes. The WHO guideline release has reinforced that infertility care should address major modifiable risk factors through lifestyle interventions while recognizing the emotional toll of infertility and the need for psychosocial support [1,7]. At the same time, the evidence base remains heterogeneous in stress definitions, biological readouts, and study designs, and the field continues to evolve toward integrated models that can connect psychosocial exposures with clinically meaningful reproductive endpoints.

Oxidative stress mechanisms and redox-targeted adjuncts in male infertility, as well as empirical management approaches that include lifestyle modification, have been reviewed previously [7]. However, a focused synthesis that places stress biology upstream of redox dysregulation remains limited. The present review addresses this gap by examining psychological stress as an initiating exposure and by integrating mechanistic pathways through which hypothalamic–pituitary–adrenal activation and sympathetic signalling, immune–inflammatory programs, and mitochondrial bioenergetic perturbation converge on oxidative stress within the testes, epididymis, and seminal compartment. In addition, the review integrates evidence published between 2023 and 2025, including recent systematic syntheses and guideline updates. Emerging experimental studies are also reviewed, indicating that paternal stress may be accompanied by changes in sperm epigenetic signatures, particularly small non-coding RNAs/microRNAs, with plausible biological relevance beyond fertilization. This synthesis complements existing antioxidant- or lifestyle-focused reviews by clarifying the stress–oxidative interface and delineating current translational priorities.

This review synthesizes current evidence at the interface of psychological stress, oxidative stress, and male infertility. It integrates mechanistic plausibility with human and experimental data, considers how stress-related behaviors and circadian disruption may amplify oxidative burden, and identifies implications for counseling and multidisciplinary care. Finally, it highlights key methodological gaps and research priorities required to strengthen causal inference and to translate emerging biomarkers and intervention strategies into patient-centred, evidence-based fertility care.

## 2. Methods and Literature Search Strategy

This manuscript is a narrative review. To enhance methodological transparency and reproducibility, a targeted literature search was conducted in PubMed/MEDLINE, Embase, Web of Science Core Collection, and the Cochrane Library from database inception to 31 December 2025, with the intent to capture key evidence published in 2023–2025, including recent guidelines and meta-analyses. Search strings combined concepts related to (1) male fertility and semen quality (e.g., male infertility, subfertility, semen, sperm, spermatogenesis, sperm DNA fragmentation), (2) psychological stress and related constructs (e.g., stress, perceived stress, psychosocial stress, anxiety, depression, distress, sleep disturbance/insomnia, shift work, quality of life), and (3) biological mediators and downstream pathways relevant to fertility impairment (e.g., cortisol, glucocorticoids, hypothalamic–pituitary–adrenal (HPA) axis, catecholamines/sympathetic activity, oxidative stress, reactive oxygen species, oxidation–reduction potential, inflammation, mitochondrial dysfunction, epigenetic programming). The electronic search was supplemented by manual screening of reference lists from included articles and from recent systematic reviews/meta-analyses and major clinical guidance documents (e.g., WHO infertility guidance; American Urological Association (AUA) and American Society for Reproductive Medicine (ASRM) male infertility guideline update; European Association of Urology (EAU) male sexual and reproductive health guideline update) to identify additional relevant primary studies.

Eligibility prioritized human clinical evidence in adult men, including randomized controlled trials, interventional studies, and well-designed observational cohorts assessing associations between stress-related exposures or interventions and fertility-relevant outcomes (e.g., semen parameters, sperm DNA damage/chromatin integrity, reproductive hormones, and pregnancy and assisted reproductive technology (ART) outcomes). Systematic reviews, meta-analyses, and clinical guidelines were used to contextualize the evidence base and to help identify landmark primary studies; however, evidence was synthesized qualitatively and no formal risk-of-bias grading or quantitative meta-analysis was undertaken for this review. Translational, animal, and in vitro studies were included selectively when they clarified biological plausibility or mechanistic pathways linking stress mediators to testicular function, oxidative stress, inflammation, or germline epigenetic changes. Studies without fertility-relevant outcomes, studies in pediatric populations, and reports lacking sufficient methodological detail were excluded.

## 3. Psychological Stress and Oxidative Stress: Molecular Mechanisms

### 3.1. Stress Physiology and Neuroendocrine Responses

Psychological stress initiates a cascade of neuroendocrine responses that can perturb the reproductive hormonal axis and cellular redox balance. The immediate “fight or flight” response is mediated by the sympathoadrenal system, releasing catecholamines (epinephrine and norepinephrine) that acutely raise heart rate, blood pressure, and metabolic activity [8,9]. Concurrently, activation of the HPA axis is initiated when the hypothalamus releases corticotropin-releasing hormone (CRH), which stimulates pituitary secretion of adrenocorticotropic hormone (ACTH) and, in turn, promotes adrenal glucocorticoid production, predominantly cortisol [8,9]. Acute activation of these stress pathways is adaptive and typically transient. However, chronic stress may contribute to persistently elevated cortisol and sympathetic output, which have deleterious effects on the male reproductive system [10].

Glucocorticoids can suppress the hypothalamic–pituitary–gonadal (HPG) axis at multiple levels. Cortisol and CRH both inhibit gonadotropin-releasing hormone (GnRH) release, thereby reducing luteinizing hormone (LH) and follicle-stimulating hormone (FSH) secretion and ultimately lowering testosterone production in Leydig cells [11,12,13]. Chronic high cortisol levels are believed to induce apoptosis in testicular cells—including germ cells, Sertoli cells, and Leydig cells—via glucocorticoid receptor-mediated mechanisms [14,15,16,17]. Indeed, preclinical studies show that experimentally stressed rats exhibit elevated intratesticular cortisol accompanied by significant loss of germ cells and Leydig cells due to apoptosis [18,19,20]. Prolonged stress also upregulates gonadotropin-inhibitory hormone (GnIH), a hypothalamic neuropeptide that directly suppresses GnRH and gonadotropin release. Animal studies demonstrate that chronic stress increases GnIH secretion via cortisol’s action on GnIH neurons and via stress-triggered norepinephrine signaling [21,22,23]. The net effect is an “anti-gonadal” endocrine milieu, characterized by attenuated pulsatile LH secretion and reduced testosterone output, with downstream consequences for spermatogenesis and sperm maturation [24,25].

Beyond endocrine perturbations, psychological stress can modify sexual behavior and function in ways that may compromise fertility. Men exposed to chronic stress commonly report diminished libido and a higher burden of sexual dysfunction, including erectile or ejaculatory difficulties [26,27]. Some of these effects may be mediated by suppression of the gonadal axis with consequent reductions in testosterone, whereas others appear to arise from psychological drivers such as anxiety and performance-related stress, together with stress-induced autonomic dysregulation. For instance, heightened sympathetic outflow can promote testicular vasoconstriction, thereby diminishing testicular perfusion and oxygen delivery [28,29].

This stress-related vasoconstriction, together with attenuated LH-driven testosterone production, fosters a milieu that is suboptimal for spermatogenesis [30]. Notably, even short-lived acute stress may exert measurable effects. In one study, the heightened anxiety associated with providing a semen sample during assisted reproduction was linked to a 39% reduction in sperm concentration and a 48% reduction in motility on the day of oocyte retrieval [31]. Collectively, these observations indicate that stress responses, whether acute or sustained, can rapidly influence sperm output and quality through convergent endocrine and autonomic mechanisms [32].

### 3.2. Induction of Oxidative Stress by Psychological Stress

Psychological stress is closely linked to oxidative stress through multiple biological pathways [33]. Excess glucocorticoid exposure can exert pro-oxidant effects [34]. Cortisol reshapes cellular metabolism and may disrupt mitochondrial function, thereby increasing reactive oxygen species (ROS) generation during ATP production [35]. Spermatozoa are especially susceptible to mitochondrial ROS because the midpiece contains a high density of mitochondria that power motility [36,37]. Under stress, elevated cortisol appears to preferentially perturb sperm mitochondrial function and promote ROS generation, as supported by recent work linking higher free cortisol levels with increased sperm ROS and reduced motility [38,39]. In addition, prolonged glucocorticoid exposure may weaken antioxidant defenses by downregulating antioxidant enzymes through glucocorticoid-responsive transcriptional regulation, although the precise molecular details remain to be fully clarified [40,41]. In rodent models, chronic restraint or social stress significantly depletes testicular antioxidant enzymes such as superoxide dismutase (SOD), catalase (CAT), glutathione peroxidase (GPx), and glutathione transferases, concurrently raising markers of lipid peroxidation like malondialdehyde (MDA) [42]. For example, one study subjected rats to 60 days of intermittent stress and observed a significant increase in testicular MDA with a corresponding >50% decrease in SOD, CAT, and GPx activities compared to controls [43]. These oxidative changes were accompanied by extensive germ cell loss, linking chronic stress to oxidative damage in the testes [43].

Psychological stress-induced sympathetic activation can amplify oxidative stress via adrenergic regulation of cellular metabolism and immune function [44]. Norepinephrine can reprogram immune-cell trafficking and effector programs in a receptor- and context-dependent manner, and in some cell types, it can directly enhance ROS production through NADPH oxidase signaling [45]. Adrenergic signaling also modulates mitochondrial oxidative phosphorylation, while stress-related hyperglycemia further increases mitochondrial ROS pressure [46,47]. In the male reproductive tract, catecholaminergic input to the testis is well documented; adrenergic signaling is linked to alterations in testicular microcirculation/oxygenation and can promote local inflammatory and oxidative injury pathways [48,49].

One of the most compelling mechanistic links between psychological stress and ROS generation involves immune activation and inflammation. Although acute stress may transiently suppress selected immune functions, chronic stress is more consistently associated with persistent, low-grade inflammatory activity [50,51]. Psychological stress increases circulating pro-inflammatory cytokines, including interleukin 6 (IL-6) and tumor necrosis factor alpha (TNF-α), and can promote activation of tissue macrophages and other immune effector cells [52,53].

Within the male reproductive tract, stress has been associated with prostatitis and leukocytospermia, defined as increased leukocyte counts in semen, even when overt infection is not demonstrated [54]. The concept of stress prostatitis has been introduced to describe prostatitis symptoms that are triggered or exacerbated by psychological stress [55]. Activated leukocytes, particularly neutrophils and macrophages, within the prostate, epididymis, and seminal fluid can generate substantial quantities of ROS [56,57]. Experimental estimates indicate that activated leukocytes may produce ROS at levels up to one thousand-fold higher than in non-activated conditions. As a result, leukocytospermia is a well-recognized driver of oxidative stress in semen and has been linked to impaired sperm motility and compromised DNA integrity [58,59].

**Figure 1:**
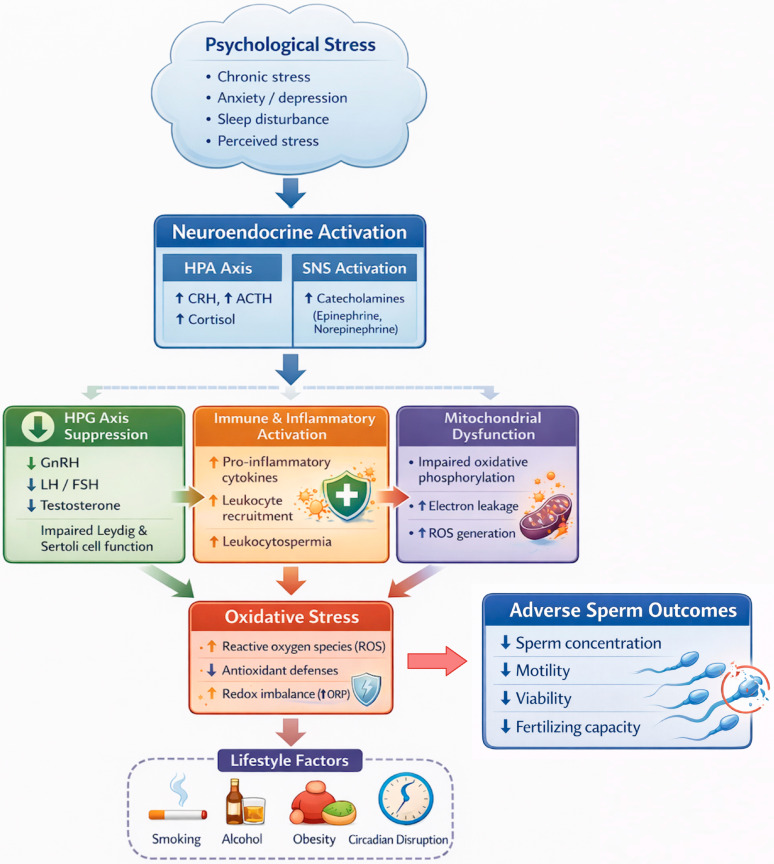
Psychological stress promotes oxidative stress and adverse sperm outcomes via neuroendocrine, immune–inflammatory, and mitochondrial pathways. Stress constructs reflected in the clinical and experimental literature include general perceived stress or psychological distress, infertility and treatment-related distress, and sleep or circadian disruption. Psychological stress activates the hypothalamic–pituitary–adrenal (HPA) axis and the sympathetic nervous system (SNS), leading to downstream suppression of the hypothalamic–pituitary–gonadal (HPG) axis, immune and inflammatory activation, and mitochondrial dysfunction. These converging mechanisms increase reactive oxygen species (ROS) and reduce antioxidant defenses, resulting in redox imbalance (including increased oxidation–reduction potential, ORP) and impaired sperm quality (e.g., reduced concentration, motility, viability, and fertilizing capacity). Lifestyle factors (smoking, alcohol use, obesity, and circadian disruption) may exacerbate oxidative stress and amplify these effects. Evidence for reversibility is context dependent. In experimental models, several redox and semen abnormalities are at least partially reversible after stress cessation and or targeted interventions, whereas prolonged exposure can result in incomplete recovery. Abbreviations: ACTH, adrenocorticotropic hormone; CRH, corticotropin-releasing hormone; FSH, follicle-stimulating hormone; GnRH, gonadotropin-releasing hormone; HPA, hypothalamic–pituitary–adrenal; HPG, hypothalamic–pituitary–gonadal; LH, luteinizing hormone; ORP, oxidation–reduction potential; ROS, reactive oxygen species; SNS, sympathetic nervous system. Created in BioRender. Kaltsas, A. (2025) https://BioRender.com/3nl28jx, accessed on 18 December 2025.

Stress-related inflammation may therefore initiate a self-reinforcing cascade in which psychological stress promotes inflammatory signaling within reproductive tissues, inflammation facilitates leukocyte recruitment, and recruited leukocytes generate ROS that injure sperm [60]. Consistent with this framework, prostate-derived leukocytes have been implicated in ROS-mediated reductions in sperm motility, and leukocyte concentrations exceeding 1 × 10^6^ per milliliter in semen are generally considered pathological [56]. Stress may further augment leukocyte-rich prostatic secretions through neuroimmune pathways, thereby providing a biologically plausible route by which psychological stress is transduced into oxidative damage within the seminal microenvironment [61,62]. Figure 1 summarizes the major neuroendocrine, autonomic, immune–inflammatory, mitochondrial, and lifestyle-related pathways through which psychological stress promotes oxidative stress and contributes to adverse sperm outcomes.

## 4. Oxidative Stress in the Male Reproductive System and Impact on Sperm

Oxidative stress is widely recognized as a major mediator of male infertility, in which injury to sperm structure and function represents a central pathological process [63]. Spermatozoa are intrinsically vulnerable to oxidative damage because their plasma membranes are enriched in polyunsaturated fatty acids and their cytosolic antioxidant capacity is limited because of minimal cytoplasmic volume [64]. When ROS generation overwhelms antioxidant protection, a cascade of detrimental effects follows [65].

### 4.1. Lipid Peroxidation

ROS, particularly hydroxyl and peroxyl radicals, attack unsaturated fatty acids within the sperm plasma membrane. This initiates lipid peroxidation chain reactions and generates end products such as MDA, which can form adducts and promote cross-linking of proteins and nucleic acids [66]. Lipid peroxidation compromises membrane fluidity and structural integrity, thereby reducing sperm viability and impairing motility [67]. Effective flagellar propulsion depends on an adequately fluid membrane and intact ion channel function, and peroxidative injury can rigidify the membrane and disrupt axonemal protein activity, culminating in asthenozoospermia [68]. Consistent with this mechanism, higher seminal MDA concentrations have been repeatedly associated with lower sperm motility and vitality [69,70].

### 4.2. DNA Damage

ROS can penetrate the sperm nucleus or arise from intracellular sources and induce DNA strand breaks, along with oxidative base lesions such as 8-oxoguanine [64,71]. Under conditions of oxidative stress, the sperm DNA fragmentation index (DFI) is frequently increased [72]. Spermatozoa possess only a limited capacity for DNA repair because their chromatin is highly condensed and they are transcriptionally inactive, so oxidative lesions may persist and impair the integrity of the paternal genome delivered at fertilization [73]. Elevated sperm DNA fragmentation has been associated with reduced fertilization rates, poorer embryo development, increased miscarriage risk, and adverse health outcomes in offspring [74]. In clinical cohorts, higher seminal ROS levels have been linked to greater sperm DNA fragmentation and lower pregnancy success [75,76]. Across diverse etiologies, including infection, smoking, and environmental exposures, oxidative stress is considered a dominant contributor to sperm DNA damage in infertile men and has been estimated to account for the majority of observed DNA fragmentation [77,78].

### 4.3. Protein Oxidation and Enzyme Inactivation

ROS can oxidize amino acid side chains and thereby alter or inactivate proteins essential for sperm competence [64]. Targets include axonemal proteins that drive progressive motility, surface receptors implicated in zona pellucida binding and gamete interaction, and enzymes required for energy production [79]. Oxidative injury within the midpiece can also compromise mitochondrial proteins, diminishing ATP generation and further impairing motility [80]. In parallel, oxidative stress can trigger caspase-dependent signaling and induce apoptosis-like alterations in sperm, often described as abortive apoptosis, in which spermatozoa exhibit features such as phosphatidylserine externalization and chromatin condensation despite an inability to complete canonical cell death programs [81]. Collectively, these processes reduce the proportion of functionally competent spermatozoa.

### 4.4. Apoptosis of Germ Cells

Within the testes, oxidative stress can disrupt spermatogenesis by injuring developing germ cells and impairing Sertoli cell support [82,83]. Excess ROS in the intratesticular microenvironment promotes apoptosis of spermatocytes and spermatids, which may present clinically as oligozoospermia and, in more severe cases, azoospermia [84]. Evidence from experimental models supports this concept, as rodents exposed to chronic stress demonstrate increased numbers of terminal deoxynucleotidyl transferase dUTP nick-end labeling (TUNEL)-positive germ cells within seminiferous tubules, together with reduced sperm output [85]. With sustained exposure, oxidative injury may also contribute to structural remodeling of the testis, including fibrotic change and progressive atrophy, thereby further limiting spermatogenic capacity [86].

In light of these mechanisms, it is plausible that oxidative stress contributes substantially to idiopathic male infertility [87]. Reported estimates suggest that a considerable subset of men classified as having idiopathic infertility exhibit increased seminal oxidant activity or reduced antioxidant defenses [88]. In clinical practice, oxidative stress-related infertility is increasingly evaluated using assays of oxidation–reduction potential, together with complementary endpoints such as oxidative DNA damage markers and sperm DNA fragmentation [72]. Across studies, men with impaired fertility more often show higher oxidative biomarkers and lower antioxidant capacity than fertile controls [89,90]. The observation that antioxidant interventions and targeted lifestyle modification frequently improve semen parameters provides additional, albeit indirect, support for oxidative damage as a relevant component of the pathophysiology [5,91].

## 5. Evidence from Human Studies

Epidemiological and clinical studies in humans have investigated the link between psychological stress and male fertility parameters, often with a focus on markers of oxidative stress. Overall, the evidence suggests a deleterious impact of stress on semen quality, although results have varied somewhat depending on study design and stress measures [66]. Most human studies are cross-sectional and susceptible to reverse causality and residual confounding; therefore, causal inference remains limited. A summary of representative human studies is provided in Table 1.

### 5.1. Cross-Sectional Studies of Stress and Semen Quality

Several studies have examined associations between men’s self-reported stress, typically assessed with validated questionnaires, or exposure to stressful life events, and semen quality. In an early study, Hjollund and colleagues in 2004 reported that, among healthy Danish men, higher perceived psychological stress was not clearly associated with standard semen parameters, yet greater stress was accompanied by a tendency toward reduced fecundability, particularly among men with already suboptimal semen quality [101]. By contrast, Janevic and colleagues in 2014 found linear relationships between work-related stress and broader life stress and poorer semen quality in a clinical sample, with men reporting the highest stress demonstrating significantly lower sperm concentration, fewer morphologically normal spermatozoa, and a higher prevalence of oligospermia than men reporting lower stress [96]. These differences suggest that the operationalization of stress is consequential, and that subjective appraisal may be more informative than the occurrence of external events alone [96]. Consistent with this view, Nordkap and colleagues in 2020 evaluated perceived stress, stressful life events, and stress symptoms in more than 1300 young men and observed that perceived stress showed the most consistent associations with impaired semen parameters [94]. Men with the highest perceived stress scores had, on average, 38% lower sperm concentration and 42% lower total sperm count than men with the lowest scores, and they also exhibited significantly lower proportions of motile sperm [94]. In the same study, the burden of major stressful life events, such as job loss or bereavement, was not associated with semen quality, reinforcing the relevance of internalized stress. Higher perceived stress was additionally accompanied by an approximately 25% increase in serum follicle-stimulating hormone (FSH), consistent with reduced spermatogenesis and compensatory pituitary drive. Importantly, these associations remained significant after adjustment for smoking, alcohol intake, body mass index, and related lifestyle factors, suggesting an effect not fully explained by adverse health behaviors [94].

A study of 423 men by Bräuner and colleagues in 2020 compared men diagnosed with male-factor infertility with proven-fertile controls, defined as partners of pregnant women [95]. The investigators hypothesized that infertile men would report higher psychological stress. However, the groups did not differ significantly in general stress symptom scores or indices of chronic stress. Although a greater proportion of infertile men had experienced at least one major stressful life event in the recent period, with 50% compared with 36% in controls, and more frequently reported relationship difficulties, overall levels of psychological stress were comparable between fertile and infertile participants [95]. In addition, semen parameters in this cohort did not show a clear association with questionnaire-derived stress measures. The authors highlighted the unexpected nature of these null findings and proposed that incorporating biochemical indicators of stress, such as cortisol, or using more sensitive psychometric instruments might be required to detect subtler effects. These results also emphasize that infertility-related distress can complicate inference in clinical samples, since infertility itself constitutes a potent stressor and may obscure pre-existing differences [95].

Despite heterogeneity across individual studies, the broader literature, including systematic reviews, generally supports an inverse association between psychological stress and semen quality. A meta-analysis by Li and colleagues in 2011, which pooled data across multiple studies, concluded that psychological and lifestyle-related stressors were associated with a higher likelihood of abnormal semen parameters [99]. Overall, the most consistent adverse associations involve sperm concentration, total sperm count, and motility, whereas findings for morphology have been less uniform [99]. Emerging evidence also suggests potential links with sperm DNA integrity. Recent studies reported that higher depressive symptom scores were associated with increased sperm DNA fragmentation, with the strongest effects observed among men who also reported poor sleep habits [103,104,105].

### 5.2. Mechanistic and Biomarker Studies

Some clinical investigations have evaluated oxidative stress-related signatures in men reporting elevated psychological stress. In a cross-sectional study, Wang and colleagues in 2025 identified molecular alterations in sperm associated with higher depression, anxiety, and stress scores, including dysregulation of the mitochondrial pyruvate dehydrogenase kinase (PDK) and pyruvate dehydrogenase complex (PDC) axis that was linked to impaired sperm motility [92]. This observation supports the premise that psychological stress may remodel sperm bioenergetics in a manner that favors inefficient mitochondrial respiration and increased reactive oxygen species generation [106]. Complementary studies assessing seminal redox status have reported higher seminal ROS levels and reduced antioxidant reserves, such as diminished glutathione, among men with higher anxiety or stress scores compared with low-stress counterparts [100,107]. In the context of infertility care, clinically significant anxiety or depressive symptoms have also been associated with greater oxidative DNA damage in sperm, reflected by increased 8-hydroxy-2-deoxyguanosine (8-OH-dG) levels [93,98].

### 5.3. Stress from Infertility and Treatment

Infertility itself can be a substantial psychological stressor for men, raising the possibility of a bidirectional association between distress and reproductive function. Systematic analyses and recent reviews indicate that men diagnosed with infertility—particularly male-factor infertility—frequently experience elevated symptoms of anxiety, depression, and reduced quality of life, and that infertility care providers should recognize men’s distinct psychosocial support needs [3,108]. Directionality remains challenging to disentangle because distress may both precede and follow adverse reproductive outcomes; notably, longitudinal ART data suggest that treatment failure can predict subsequent psychological distress even when pretreatment distress does not predict treatment failure [109].

Longitudinal semen sampling during IVF further illustrates this complexity: semen quality often changes between a baseline sample and the day of oocyte retrieval, with some evidence linking specific stress appraisals (e.g., pressure around producing the sample) to poorer semen parameters, while other studies report similar semen changes that are not explained by questionnaire-derived chronic stress [97,102]. Taken together, these data support a multifactorial, potentially reciprocal interaction between stress and male fertility. Clinically, integrating psychosocial screening and supportive interventions during infertility care is increasingly viewed as relevant—not only to patient well-being but also because fertility-related stress has been associated with poorer treatment outcomes and because structured programs that reduce infertility stress and promote health behaviors can coincide with improvements in semen parameters or oxidative stress biomarkers [110,111,112].

### 5.4. Psychological Stress Constructs and Measurement Instruments

Human studies quantify psychological stress using nonequivalent constructs and instruments, which contributes to between-study heterogeneity and complicates synthesis. Perceived stress, defined as the subjective appraisal of stress in daily life, is commonly assessed with brief questionnaires such as the Perceived Stress Scale [113]. Broader psychological distress has also been operationalized as a composite construct that generates separate depression, anxiety, and stress domain scores, most often with multidomain instruments such as the Depression Anxiety Stress Scales [114]. Anxiety is frequently measured with state–trait instruments such as the State–Trait Anxiety Inventory, which distinguishes situational anxiety from dispositional anxiety and may therefore capture different exposure windows [115]. In infertility settings, studies have used combined symptom scales such as the Hospital Anxiety and Depression Scale to screen for anxiety and depressive symptoms, whereas depressive symptom burden has also been captured with dedicated inventories including the Beck Depression Inventory [116,117].

A second category comprises context-specific stress, particularly infertility-related distress. Infertility-specific instruments such as the Fertility Problem Inventory quantify domains that generic stress scales may miss, including relationship strain, sexual concerns, and social pressure, and may therefore better reflect exposures directly linked to infertility and treatment experiences [118]. A third category includes objective stress exposure assessed by life event inventories or checklists that count major stressful events such as bereavement or job loss [119]. Finally, several studies capture acute situational stress tied to time-limited events, including examination periods and stress around semen collection in assisted reproduction settings [120,121]. Acute stress measured near semen collection has been associated with short-term decrements in sperm concentration and motility in early assisted reproduction cohorts [102].

This variability in stress measurement can influence effect estimates through several mechanisms. First, different instruments capture partly distinct constructs, so exposure misclassification is likely when results are compared across studies that label their exposure as stress but measure non-overlapping domains. Second, perceived stress scales may show stronger associations with reproductive outcomes than life event counts because appraisal-based measures better reflect chronic psychophysiological load and coping, while event lists do not capture intensity, duration, or personal meaning [94,96]. Consistent with this, Nordkap and colleagues reported inverse associations between perceived stress and semen quality, while major life events were not associated with the same outcomes [94]. Third, clinical samples are vulnerable to reverse causality because infertility itself can increase anxiety and depressive symptoms, which may inflate correlations between symptom scales and semen parameters or obscure pre-existing differences between fertile and infertile men [4,95,122]. Fourth, the choice of psychometric tool can change prevalence estimates and group classification, as shown in meta-analytic work where depression prevalence in infertile men varied materially across instruments [122]. Overall, this exposure heterogeneity limits causal inference and can generate inconsistent findings even when the underlying biology is similar [123].

Future studies would benefit from greater standardization, including a core set of validated measures aligned to specific constructs, repeated assessments across the spermatogenic window, and complementary objective indicators such as physiological stress biomarkers when feasible [95,123].

## 6. Evidence from Animal and Experimental Studies

Animal models have been invaluable in demonstrating the causal effects of psychological stress on male reproductive function and the mediating role of oxidative stress. Unlike human studies, animal experiments can apply defined stressors and directly measure outcomes in a controlled setting [124]. A summary of representative animal and experimental models is provided in Table 2.

### 6.1. Rodent Models of Chronic Stress

Rodents subjected to chronic stress paradigms reliably show impairments in spermatogenesis and sperm quality, along with evidence of oxidative damage in reproductive organs. For example, chronic restraint stress or unpredictable stress for 4–8 weeks in rats results in decreased testis weight, reduced sperm counts and motility, and increased abnormal sperm morphology compared to unstressed controls [43]. Stress-exposed rats also exhibit elevated apoptosis in testicular germ cells and reduced serum testosterone, mirroring the endocrine disruption seen in stressed humans [43]. Crucially, these effects are strongly linked to oxidative stress markers. As mentioned earlier, Nirupama et al. showed that rats undergoing 60 days of combined restraint and swim stress had significantly lower antioxidant enzyme levels and higher MDA in the testes, indicating considerable oxidative injury [43]. Even after a 4-month recovery period post-stress, many oxidative parameters and sperm defects did not fully normalize, suggesting that chronic stress can inflict long-lasting or irreversible oxidative damage to the spermatogenic apparatus [43]. Another study found that chronic intermittent cold-water stress in rats similarly raised testicular MDA and decreased SOD/CAT, correlating with poor sperm motility and fertility outcomes [42].

A recent study by Yang and colleagues in 2025 offers direct experimental evidence that psychological stress, in the absence of concurrent physical stressors, can induce oxidative stress within the testes and adversely affect male reproductive behavior. Male rats exposed to chronic psychological stress through behavioral paradigms exhibited significant increases in testicular MDA and nitric oxide (NO) levels, accompanied by structural disruption of the seminiferous tubules [125]. The stressed animals also demonstrated attenuated sexual behavior, reflected by reduced female preference and fewer mating attempts, thereby linking stress not only to testicular and spermatogenic pathology but also to diminished mating performance. The authors interpreted these reproductive impairments as plausibly mediated by oxidative injury within testicular tissue [125]. This interpretation is further supported by earlier experimental work showing that antioxidant administration in chronically stressed rodents can partially restore testosterone concentrations and improve sperm counts, suggesting a causal role for oxidative mechanisms in stress-related reproductive dysfunction [126,127]. Nevertheless, interventional evidence remains limited, and additional rigorously designed studies are required to confirm the magnitude, consistency, and reproducibility of these effects.

Chronic unpredictable mild stress paradigms in mice, which are widely used to model a depression-like phenotype, have likewise been associated with reduced sperm counts and impaired fertilizing capacity, together with evidence of increased oxidative stress in the testes and epididymis [129,135,136]. Experimental work further suggests that stress can compromise the integrity of the blood–testis barrier through oxidative mechanisms, with elevated reactive oxygen species linked to depletion of tight junction proteins and increased barrier permeability [128,129]. Notably, antioxidant repletion and stress cessation with adequate recovery have been reported to restore barrier integrity and improve sperm output, supporting the concept that a proportion of oxidative injury remains reversible when the stressor is mitigated in a timely manner [130].

### 6.2. Molecular Pathways and Transgenerational Effects

Animal models extend beyond conventional endpoints such as sperm concentration and bulk reactive oxygen species measurements, enabling detailed interrogation of stress-related molecular alterations within the male reproductive tract. In chronically stressed rodents, testicular expression of pro-oxidant mediators, including inducible nitric oxide synthase, has been reported to increase, whereas the expression of antioxidant and cytoprotective programs, including genes regulated through the nuclear factor erythroid 2-related factor 2 (Nrf2) pathway, appears attenuated [130]. Together, these shifts provide a coherent transcriptional signature consistent with a stress-induced pro-oxidant state. In parallel, evidence indicates that psychological stress can activate endoplasmic reticulum (ER) stress responses within the testes, a process that may interact with oxidative stress to disrupt proteostasis, impair cellular function, and promote germ cell injury [131,137].

A particularly active area of investigation concerns the possibility that paternal stress influences offspring phenotypes through epigenetic remodeling of sperm. Chronic stress exposure in male mice and zebrafish has been shown to alter sperm microRNA profiles and other epigenetic features, changes that can be conveyed to the embryo and shape offspring stress responsivity and aspects of neurodevelopment [132]. In mice, paternal stress has been associated with specific alterations in sperm microRNAs, and experimental introduction of these microRNAs into zygotes has recapitulated selected offspring traits observed after paternal stress exposure, including altered HPA axis regulation [133]. In zebrafish, chronic unpredictable stress in males has been linked to offspring with modified expression of genes involved in stress signaling and developmental pathways [134,138]. Although such transgenerational effects do not directly constitute infertility outcomes, they highlight that stress can modify sperm at the molecular level with biological consequences that extend beyond fertilization. They also raise the possibility that some heritable signatures of paternal stress may be connected to oxidative injury, given the capacity of ROS to influence nucleic acids and their regulatory landscapes [139]. This remains an emerging field, but it reinforces the broader clinical implication that mitigating psychological stress in prospective fathers may support not only reproductive potential but also downstream offspring health.

## 7. Behavioral and Lifestyle Pathways Linking Stress to Male Infertility

Psychological stress rarely exists in isolation; it often leads to or coexists with lifestyle behaviors that can themselves impair male fertility, frequently through oxidative stress mechanisms [7,140,141]. Several exposures discussed below exert stress-independent, pro-oxidant, and endocrine effects. In the context of this review, the clinically relevant question is where psychological stress increases exposure likelihood, intensity, or persistence, and where stress-related neuroendocrine activation amplifies oxidative and inflammatory consequences within the testes, epididymis, and seminal compartment. This framing helps distinguish confounding lifestyle correlates of distress from pathways in which stress plausibly worsens redox injury through maladaptive coping behaviors.

### 7.1. Smoking and Tobacco Use

Tobacco smoking is a well-established modifiable risk factor for male subfertility and has been linked to poorer semen quality and greater sperm DNA damage. Psychological distress and perceived stress can promote smoking persistence and relapse, suggesting an indirect pathway by which stress may worsen reproductive outcomes via increased tobacco exposure [142,143]. Cigarette smoke contains numerous oxidants and toxicants, including polycyclic aromatic hydrocarbons (PAHs) and heavy metals, which can increase reactive oxygen and nitrogen species generation and deplete systemic antioxidants [64,144]. In clinical studies, men who smoke exhibit poorer semen parameters and higher levels of sperm DNA fragmentation than non-smokers [145,146], and recent meta-analytic evidence indicates that smoking is among the lifestyle exposures most strongly associated with elevated sperm DNA fragmentation [147]. In parallel, seminal biomarker profiles indicate a shift toward a more pro-oxidant milieu, characterized by increased leukocyte burden and reactive oxygen species generation together with diminished antioxidant capacity [148,149]. Tobacco-derived PAHs can form bulky DNA adducts in spermatozoa, including benzo[a]pyrene diol epoxide adducts, thereby providing a mechanistic link between smoking exposure and sperm DNA damage that may be conveyed to the embryo at fertilization [150]. Encouragingly, semen parameters may improve within months after smoking cessation, supporting cessation as a core component of male infertility care; cessation support may be especially important in men with sustained stress, in whom relapse risk is higher and stress-driven smoking may perpetuate oxidative burden [151,152]. Contemporary guideline recommendations similarly emphasize counseling on smoking cessation as part of lifestyle management in infertile men [2,153]. Nicotine exposure should also be considered beyond combustible cigarettes. Nicotine delivered through electronic cigarettes, heated tobacco products, oral nicotine pouches, smokeless tobacco, and nicotine replacement therapies can activate nicotinic cholinergic receptors across tissues, alter autonomic and hormonal signaling, and contribute to redox imbalance. Evidence indicates that nicotine from diverse delivery tools modulates autonomic and endocrine pathways in ways that may favor oxidative stress and thus may represent an additional redox-relevant exposure in men undergoing fertility evaluation [154].

### 7.2. Alcohol and Substance Use

As with tobacco use, alcohol consumption may increase during periods of sustained psychological stress and can function as a maladaptive coping strategy. Excessive alcohol exposure can compromise male reproductive health through convergent endocrine and oxidative pathways. Ethanol is oxidized to acetaldehyde, and microsomal metabolism mediated by cytochrome P450 2E1 increases reactive oxygen species generation, thereby promoting oxidative stress and inflammation while consuming antioxidant reserves such as glutathione [155]. These redox disturbances can impair testicular function, including Leydig cell steroidogenesis and Sertoli cell support of germ cell development, and may also adversely influence the post-testicular maturation milieu, with downstream consequences for sperm quality and fertilizing capacity [156].

Human evidence broadly supports an adverse association that appears most consistent at higher exposure levels. In a contemporary meta-analysis, alcohol intake was associated with reduced semen volume and lower seminal antioxidant enzyme activity, together with decreases in testosterone and gonadotropins, whereas pooled associations with sperm concentration, motility, morphology, and sperm DNA fragmentation were heterogeneous across studies and exposure definitions [157]. In parallel, studies focused specifically on heavy consumption report poorer conventional semen parameters and higher chromatin immaturity and sperm DNA fragmentation among drinkers compared with abstainers, supporting a plausible oxidative–genotoxic pathway [158]. Recent clinical syntheses emphasize a dose-dependent pattern in which chronic high intake, including daily consumption above approximately 50 to 60 g of ethanol, and binge drinking are most reproducibly linked to impaired semen outcomes and disruption of the hypothalamic–pituitary–gonadal axis, with at least partial recovery documented after abstinence in selected cohorts [159]. At the same time, clinical findings at low-to-moderate intake can be inconsistent, and at least one large cohort in fertility treatment did not observe an association between overall alcoholic beverage intake and standard semen parameters [160]. In addition, alcohol can disrupt sleep quality and nutritional status, indirectly increasing oxidative burden and further undermining reproductive function [161].

Overall, stress-related heavy drinking is best viewed as a modifiable amplifier of oxidative and endocrine dysregulation rather than a necessary intermediary for alcohol associated reproductive harm [159].

### 7.3. Sleep Disturbances and Circadian Disruption

Chronic psychological stress commonly manifests as insomnia, fragmented sleep, and irregular sleep timing. Sleep disturbance therefore operates as both a stress-correlated exposure and a plausibly independent driver of oxidative and inflammatory load, creating a bidirectional loop that may magnify redox injury in the male reproductive tract [162]. Sleep and circadian rhythms coordinate neuroendocrine regulation and redox homeostasis, and inadequate sleep may promote reactive species accumulation and impair redox-sensitive antioxidant responses, including pathways regulated by Nrf2 [162].

Human evidence increasingly supports an association between disturbed sleep and impaired semen quality. A systematic review and meta-analysis published in 2022 reported that sleep disorders were associated with lower total sperm count, lower sperm concentration, reduced progressive motility, and reduced normal morphology, whereas consistent differences in semen volume and reproductive hormones were not demonstrated across studies [163]. More recent population-based work strengthens the signal. A North American preconception cohort study of pregnancy planners, including 690 men contributing 1247 semen samples, found that poor sleep health, encompassing short and long sleep duration, frequent sleep trouble, and poor sleep quality, was associated with reduced sperm concentration, total sperm count, and total motile sperm count; short sleep and frequent sleep trouble were also linked to lower semen volume [164]. In clinical infertility settings, poor male sleep quality has likewise been associated with lower sperm concentration and motility and with reduced probability of partner pregnancy, while gonadotropins and testosterone did not differ materially between good and poor sleep groups in that cohort [165]. Complementing these findings, a cohort study of young men reported U-shaped associations between sleep duration and sperm chromatin immaturity assessed by high DNA stainability, together with corresponding differences in androgen biomarkers such as testosterone and free androgen index [166].

Preclinical models provide biological plausibility and suggest that a component of sleep-related reproductive impairment may be reversible. In a rat model of chronic sleep restriction, sleep loss increased permeability of the blood–testis and blood–epididymis barriers and reduced expression of tight junction proteins and androgen receptor, changes accompanied by reduced sexual behavior and fewer pregnancies; recovery sleep over several days progressively re-established fertility [167]. In a complementary continuous sleep deprivation model, male rats exhibited reduced epididymal sperm concentration and motility, reduced serum testosterone, and a segment-specific inflammatory response characterized by immune cell activation and cytokine and chemokine accumulation in the cauda epididymis; recovery sleep mitigated epididymal injury and improved sperm quality, and Mendelian randomization analyses in the same work were consistent with an association between sleep disorders and male infertility [168]. Other rodent data indicate that sleep deprivation can induce structural testicular injury with marked reductions in multiple germ and somatic cell populations, supporting a tissue-level substrate for reduced sperm output [169].

Circadian disruption related to night or rotating shift work extends this framework and may be particularly relevant when occupational strain coexists with chronic stress. A 2025 systematic review and meta-analysis found that night shift work was associated with lower sperm counts compared with non-shift workers, while hormone findings were heterogeneous and limited by study quality [170]. In men undergoing fertility evaluation, night shift work has also been associated with higher systemic oxidative stress and lower sperm motility; a small exploratory intervention suggested antioxidant supplementation reduced oxidative stress markers in both night and day workers, with a larger reduction in night workers, although controlled trials are required to define efficacy [171]. Taken together, sleep and circadian disruption represent clinically pragmatic targets that may reduce oxidative burden, particularly in men whose sleep disruption is stress-linked [163,164].

### 7.4. Other Lifestyle Factors

Chronic psychological stress can also reshape diet, body weight, physical activity, and exposure to additional substances, creating convergent metabolic and redox pressures that may undermine male reproductive function [7,140,141]. In this domain, stress often acts upstream by promoting maladaptive eating and inactivity, while obesity and metabolic dysfunction exert strong stress-independent effects through systemic inflammation, oxidative stress, and altered sex steroid milieu [172]. Excess adiposity is consistently linked to impaired semen quality and altered reproductive hormones, and recent meta-analytic evidence indicates that elevated body mass index is associated with poorer sperm concentration, motility, and morphology, together with measurable differences in circulating testosterone and gonadotropins [173]. These associations are biologically plausible because obesity promotes systemic inflammation and oxidative stress, increases aromatization and estradiol exposure, and is often accompanied by comorbidities such as insulin resistance, diabetes, and sleep-disordered breathing, all of which can further impair the endocrine and cellular environment required for spermatogenesis [172]. Importantly, emerging interventional evidence suggests that optimizing diet and physical activity in men with obesity can yield measurable improvements in sperm quality, even when weight loss is modest. In a recent systematic review and meta-analysis of obesity interventions, lifestyle change was associated with improvements in progressive motility and normal morphology, although randomized trial data remain limited and heterogeneous [172]. The same review also highlights that surgical and pharmacologic weight loss modalities may not translate into consistent reproductive benefit, and that nutritional sufficiency and the reproductive implications of newer anti-obesity agents, including glucagon-like peptide-1 (GLP-1)-based therapies, remain insufficiently characterized for preconception counseling [172].

Physical activity represents a second domain in which stress-related behavior change may influence fertility risk. Psychological stress often reduces habitual exercise through fatigue, time scarcity, and low motivation, fostering sedentary behavior that reinforces metabolic dysfunction. In contrast, regular moderate intensity activity appears broadly favorable for male reproductive health, with recent clinical syntheses suggesting that structured exercise programs can improve key semen parameters [174]. At the same time, the relationship between exercise and reproduction is likely nonlinear, with excessive training loads and vigorous intensity activity plausibly imposing countervailing stressors through increased oxidative burden, inflammation, and heat exposure. Supporting this concept, a 2025 prospective cohort analysis reported an inverted U-shaped association in which medium levels of physical activity were linked to the most favorable semen quality, while higher intensity patterns did not confer the same benefit and were associated with less favorable profiles for selected parameters Clinically, this supports individualized counseling that distinguishes stress related inactivity from high load training as different exposure patterns with different oxidative implications [174,175].

A further concern is the use of recreational drugs and performance-enhancing substances as maladaptive coping strategies or as culturally reinforced behaviors in high stress contexts. Exogenous testosterone and anabolic androgenic steroids are particularly important because they directly suppress the hypothalamic pituitary gonadal axis, reduce intratesticular testosterone, and can precipitate severe oligozoospermia or azoospermia with testicular atrophy [176,177]. Contemporary reviews further emphasize that chronic androgen abuse can promote oxidative stress and sperm DNA damage and that recovery after cessation may be prolonged and incompletely reversible in a subset of men, sometimes requiring pharmacologic stimulation of gonadotropin signaling and assisted reproduction when fertility is time sensitive [176,177]. Beyond androgens, a recent open-access review synthesizes evidence that multiple psychoactive drugs, including opioids, stimulants, and cannabis, can impair spermatogenesis and sperm quality through mechanisms that include reactive oxygen species-dependent testicular and sperm oxidative damage, inflammatory activation, apoptosis, and suppression of the hypothalamic pituitary testicular axis [178]. Overall, diet quality, metabolic health, physical activity patterns, and substance exposures can co-occur with psychological stress and amplify oxidative and endocrine disruption through mutually reinforcing pathways, thereby shaping male reproductive potential in clinically meaningful ways.

## 8. Limitations, Clinical and Translational Implications, and Future Directions

### 8.1. Study Limitations

The literature linking psychological stress, oxidative stress, and male infertility remains constrained by measurement and design limitations. Most clinical studies quantify stress using self-reported questionnaires that capture perceived distress but do not reliably reflect biological stress physiology. As a result, exposure misclassification is likely, particularly when stress is episodic, culturally mediated, or consciously underreported. In addition, much of the human evidence is cross-sectional, often based on a single stress assessment and one semen analysis, which limits causal inference and leaves substantial vulnerability to reverse causality, since infertility itself is a potent stressor [3,108]. Within-subject variability in semen parameters further complicates interpretation, and inadequate control of confounding factors such as obesity, smoking, sleep disturbance, medications, and comorbid illness may inflate or obscure true associations.

A second major limitation is heterogeneity in oxidative stress measurement. Studies have used diverse biomarkers and platforms, including direct reactive oxygen species assays, lipid peroxidation endpoints such as malondialdehyde, total antioxidant capacity, sperm DNA fragmentation, and integrated redox measures such as ORP. These approaches differ in biological meaning, analytic performance, and susceptibility to preanalytical variation, and they lack harmonized thresholds for clinically meaningful oxidative stress. Consistent with this uncertainty, contemporary guidelines do not endorse routine reactive oxygen species testing in the standard evaluation of infertile men [2]. At the same time, newer syntheses indicate that seminal ORP is consistently higher in infertile men and is associated with poorer semen parameters, supporting its promise as a scalable marker while also high-lighting the need for standardized methodology and clinically validated cutoffs [6].

Across the evidence base, inference should be interpreted on a gradient. Experimental animal studies provide the strongest support for causality because stress exposure is assigned, timing is controlled, and tissue-level oxidative and reproductive endpoints can be directly measured. In humans, most studies remain observational and frequently cross-sectional, so associations between stress-related constructs and semen or oxidative biomarkers do not establish directionality and may be influenced by infertility-related distress, comorbid health factors, and correlated behaviors. Prospective cohorts with repeated stress and semen assessments and randomized stress reduction trials are therefore needed to strengthen causal inference and quantify clinical effect sizes.

### 8.2. Clinical Implications

Despite these constraints, the convergence of mechanistic and clinical observations supports the practical relevance of stress assessment in male infertility care, particularly in men with otherwise unexplained abnormalities. Routine psychosocial care frameworks in fertility services emphasize systematic identification of distress, provision of information, and timely referral pathways when needed [179]. Screening tools that were developed for fertility settings and have been evaluated in recent psychometric work may facilitate implementation at scale, although optimal approaches for men specifically require further validation [180,181]. Given the high prevalence of anxiety and depressive symptoms reported among infertile men, integrating mental health support into infertility pathways is clinically justifiable and may also improve adherence to lifestyle change and treatment engagement [3,4,108].

Clinical translation of oxidative stress concepts warrants caution. Major professional guidance advises clinicians to counsel patients that supplements such as antioxidants and vitamins have questionable clinical utility for male infertility, reflecting inconsistent evidence and limited ability to recommend specific agents [153]. The EAU recommends against routine antioxidant therapy for idiopathic infertility and does not recommend routine seminal reactive oxygen species assays because of limited standardization and uncertain clinical thresholds [2]. In current practice, sperm DNA fragmentation is the only oxidative-related test with clear clinical actionability supported by contemporary guidance, with selective use advised in idiopathic male infertility, recurrent pregnancy loss, and repeated ART failure, where results may change counseling and the choice or timing of interventions [2]. This position aligns with the 2024 AUA and ASRM guideline update, which also supports selective use of sperm DNA fragmentation assessment in defined scenarios rather than as a first-line test [153]. In contrast, ORP, MDA, and total antioxidant capacity (TAC) remain adjunct or research oriented measures without guideline-endorsed cutoffs for routine decision making, despite consistent associations between elevated ORP and impaired semen quality in meta analytic data [6].

Accordingly, oxidative testing is best framed as a decision tool used only when results can plausibly change management. If oxidative related abnormalities are identified, management prioritizes treatment of reversible drivers and risk factor modification, and considers a time limited antioxidant strategy only in men with documented oxidative stress and appropriate counseling regarding uncertain benefit and potential harms [182,183]. The need for restraint is supported by the SUMMER randomized clinical trial, which did not show improvement in ongoing pregnancy with antioxidant supplementation in men seeking fertility care [184]. Persistent elevation of sperm DNA fragmentation despite optimized modifiable factors, particularly after repeated ART failure, can then support escalation of sperm selection strategies and individualized discussion of surgical sperm retrieval for intracytoplasmic sperm injection (ICSI) in selected cases, consistent with a phenotype-guided approach rather than routine empiricism [2,153].

### 8.3. Translational and Public Health Implications

The stress fertility interface also has implications for prevention and health systems design. Translational efforts should focus on pragmatic screening models that combine brief validated distress instruments with targeted biomarker panels, enabling earlier identification of men at risk before prolonged spermatogenic impairment occurs. Fertility clinics represent an obvious implementation setting, but preconception care and primary care pathways may also be appropriate given the broader health relevance of semen quality. Current male infertility guidance emphasizes counseling on modifiable risk factors and recognizes male reproductive health as a window into overall health [153]. This concept is strengthened by recent population data showing that semen quality is associated with long-term health outcomes, including lifespan, supporting a broader preventive rationale for addressing chronic stress and unhealthy coping behaviors in men presenting with subfertility [153,185].

At a public health level, interventions that reduce chronic psychosocial stress and improve access to mental health resources may contribute to improved reproductive outcomes, particularly when paired with programs that address smoking, alcohol overuse, sleep disruption, and obesity. Given the documented psychological burden of infertility for men, embedding psychosocial care into routine fertility services may also reduce distress-related attrition and enhance engagement with treatment and lifestyle recommendations [3,108].

### 8.4. Gaps in Knowledge

Key uncertainties remain regarding timing, dose response, and reversibility. Human studies rarely capture stress exposure over the full spermatogenic window, limiting understanding of whether brief stressors have durable effects or whether recovery occurs reliably after stress resolution. The field also lacks consensus on which oxidative stress measures best reflect clinically meaningful sperm injury and which thresholds should trigger intervention. Although mechanistic human work is beginning to identify stress-associated molecular signatures in sperm, replication and longitudinal validation are needed before such biomarkers can guide care [92].

Transgenerational questions are also unresolved. Animal studies suggest that paternal stress can reshape sperm epigenetic profiles, but human evidence remains sparse, and the relationship between oxidative damage, epigenetic remodeling, and offspring outcomes is not yet defined. Finally, the interaction of stress with comorbid mental health disorders, sleep disturbance, occupational circadian disruption, and female partner factors remains insufficiently disentangled, particularly in assisted reproduction settings where treatment decisions and outcomes are multifactorial.

### 8.5. Future Research Directions

Progress will require study designs that match the temporal biology of spermato-genesis and the complexity of stress physiology. Prospective cohorts with repeated assessments of perceived stress, objective stress biomarkers, and serial semen analyses should be prioritized to establish temporality and to reduce exposure misclassification. Oxidative stress endpoints should be standardized, ideally incorporating both functional outcomes, such as sperm DNA fragmentation and harmonized redox measures, while simultaneously defining clinically actionable thresholds. The growing evidence base for ORP supports further evaluation of its role as a practical integrated marker, but its clinical utility must be tested in decision-oriented studies that evaluate whether biomarker-guided interventions improve patient-relevant outcomes [2,6].

Randomized trials are needed in two areas. First, stress reduction interventions should be evaluated using reproductive endpoints, including semen quality, oxidative biomarkers, and pregnancy outcomes, rather than psychological outcomes alone. Although early studies suggest that structured mind–body interventions may reduce seminal oxidative stress and improve sperm quality, the evidence remains limited and often nonrandomized [112,186]. Second, adjunctive antioxidant strategies should be tested using enrichment designs that select men with documented oxidative stress or high-risk phenotypes, given that broad supplementation in unselected fertility care populations has not improved pregnancy outcomes [153,184].

At the mechanistic level, integrated approaches combining endocrine profiling, autonomic markers, inflammatory pathways, and sperm omics should be used to map causal routes from stress to sperm dysfunction and to identify targets that are both biologically plausible and clinically druggable. In parallel, guideline development efforts that explicitly link oxidative stress testing, patient selection, and duration of therapy provide a starting framework for safer and more rational translation, but require prospective validation in diverse clinical settings [2].

## 9. Conclusions

Psychological stress may contribute to male reproductive dysfunction through convergent neuroendocrine, immune, metabolic, and behavioral pathways that culminate in oxidative stress within the testes, epididymis, and seminal compartment. Sustained activation of the HPA axis and sympathetic signaling in preclinical studies is linked to reduced gonadal endocrine support, perturbed mitochondrial and redox regulation, and heightened inflammatory activity, thereby increasing reactive oxygen and nitrogen species while weakening antioxidant defenses. The resulting oxidative imbalance is plausibly associated with lipid peroxidation, protein oxidation, and sperm DNA damage, and in controlled animal stress paradigms is accompanied by germ cell apoptosis, Sertoli and Leydig cell dysfunction, and barrier disruption, collectively reducing sperm count, motility, viability, and fertilizing capacity.

Human studies generally support associations between perceived stress, mood symptoms, sleep disruption, and poorer semen quality, although heterogeneity in stress assessment, oxidative biomarkers, and study design limits causal inference. Most human studies are cross sectional and remain vulnerable to residual confounding and reverse causality. In contrast, controlled animal models provide experimental evidence that defined stressors induce oxidative injury and reproductive impairment, with variable reversibility after stress cessation and in some paradigms partial mitigation with antioxidant interventions. Clinically, these findings justify incorporating psychosocial and lifestyle assessment into male infertility care while prioritizing modifiable factors such as smoking, alcohol, sleep, and metabolic health, while recognizing that the directionality and clinical effect sizes in humans remain to be established. Future work should focus on longitudinal cohorts aligned to spermatogenic timing, standardized redox testing, and randomized trials of stress reduction and biomarker-guided redox-targeted therapies to define reversibility, refine patient selection, and determine whether interventions improve clinically meaningful reproductive outcomes.

## Figures and Tables

**Table 1:** Key human studies linking psychological stress with semen quality and oxidative stress-related outcomes. Stress construct is specified for each study to distinguish general stress and distress, occupational stress, and infertility or ART-related situational distress. Reversibility is stated only when outcomes were reassessed after stress resolution or an intervention. Where effect size was reported in the source study, it is shown; otherwise, NR (not reported) is indicated.

Study (Reference)	Design/ Setting	Stress Measure/Exposure	Semen/Reproductive Outcomes	Oxidative Stress/Biomarker Outcomes	Main Findings
Wang et al., 2025 [92]	Cross-sectional mechanistic study (human sperm)	Depression/anxiety/stress scores (Construct: general psychological distress)	Sperm motility (focus) and quality indices	Mitochondrial bioenergetic signature (PDK–PDC axis)	Depression/anxiety/stress associated with impaired sperm quality, linked to dysregulation of the mitochondrial PDK–PDC axis (Effect size: NR; Reversibility: not assessed)
Ye et al., 2022 [93]	Cross-sectional; sperm donor candidates (*n* = 1000)	Depressive symptoms (questionnaire) (Construct: general depression symptoms)	Semen quality parameters	Oxidative stress markers; 8-OH-Dg	Depressive symptoms associated with poorer semen quality and higher oxidative stress and oxidative DNA damage (Effect size: NR; Reversibility: not assessed)
Nordkap et al., 2020 [94]	Cross-sectional; young men (*n* = 1362)	Perceived stress, stressful life events, and stress symptoms (3 scales) (Construct: general perceived stress vs. life event exposure)	Sperm concentration, total count, motility; FSH	NR	Perceived stress showed the most consistent associations: highest vs. lowest stress linked to ~38% lower concentration, ~42% lower total count, and lower motility; FSH ~25% higher; major life events not associated (Reversibility: not assessed)
Bräuner et al., 2020 [95]	Cross-sectional case–control; male-factor infertility vs. fertile controls (*n* = 423)	Stress symptoms/chronic stress indices; stressful life events (Construct: general stress symptoms and life event exposure in infertility evaluation context)	Semen parameters; testicular function markers	NR	No significant differences in general stress scores between groups; semen parameters were not clearly associated with questionnaire-derived stress measures (Reversibility: not assessed)
Janevic et al., 2014 [96]	Cross-sectional; clinical sample	Work-related and life stress (questionnaires) (Construct: occupational and general life stress)	Sperm concentration; morphology; oligospermia prevalence	NR	Higher stress associated with lower sperm concentration and fewer morphologically normal sperm; higher prevalence of oligospermia (Effect size: NR; Reversibility: not assessed)
Nouri et al., 2014 [97]	IVF patients; within-cycle comparison	Subjective/chronic stress questionnaires (Construct: general distress assessed within infertility and IVF context)	Semen quality across IVF cycle	NR	Decline in semen quality during IVF was not associated with subjective male stress (Temporal sampling within IVF cycle; Reversibility after stress cessation not assessed)
Vellani et al., 2013 [98]	Controlled study; IVF patients	State and trait anxiety (validated scales) (Construct: general anxiety assessed in infertility and ART setting)	Semen quality parameters	Sperm oxidative DNA damage marker(s) (e.g., 8-OH-dG)	Higher anxiety associated with poorer semen quality and increased oxidative DNA damage in sperm in clinical infertility context (Effect size: NR; Reversibility: not assessed)
Li et al., 2011 [99]	Systematic review and meta-analysis	Socio-psycho-behavioral stressors (pooled) (Construct: general psychosocial stressors)	Abnormal semen parameters (pooled)	NR	Overall inverse association between stressors and semen quality; most consistent adverse associations for concentration, total count, and motility; morphology findings less uniform (Reversibility: not assessed)
Eskiocak et al., 2005 [100]	Repeated-measures; healthy medical students	Exam stress (during vs. after examinations) (Construct: acute situational stress with post-stressor assessment)	NR	Seminal plasma antioxidants (glutathione; free sulfhydryl content)	Antioxidant markers decreased during exam stress and improved after the stress period (Reversibility: at least partially reversible after stress cessation)
Hjollund et al., 2004 [101]	Cross-sectional; healthy men (Denmark)	Perceived psychological stress (questionnaire) (Construct: general perceived stress)	Standard semen parameters; fecundability	NR	No clear association with standard semen parameters; stress tended toward reduced fecundability, particularly in men with suboptimal semen quality (Effect size: NR; Reversibility: not assessed)
Clarke et al., 1999 [102]	IVF patients; within-cycle sampling	Psychological stress questionnaires (Construct: infertility and ART context distress, including situational pressure around sample provision in some measures)	Semen quality across the IVF cycle	NR	Selected stress appraisals (e.g., pressure around sample provision) were linked to poorer semen parameters, but associations were heterogeneous across measures and parameters (Reversibility: not assessed)
Ragni and Caccamo, 1992 [31]	ART setting; acute situational stress	Stress/anxiety around semen collection for IVF (Construct: acute infertility and ART-related situational stress)	Sperm concentration and motility (day of retrieval)	NR	Anxiety during IVF sample provision associated with ~39% lower sperm concentration and ~48% lower motility on the day of oocyte retrieval (Reversibility: not assessed)

Abbreviations: 8-OH-dG, 8-hydroxy-2′-deoxyguanosine; ART, assisted reproductive technology; FSH, follicle-stimulating hormone; IVF, in vitro fertilization; NR, not reported; PDK, pyruvate dehydrogenase kinase; PDC, pyruvate dehydrogenase complex.

**Table 2:** Summary of key animal and experimental models linking stress exposure with oxidative injury and male reproductive impairment. For clinical interpretability, the Notes column states whether outcomes improved after stress cessation and/or after an intervention. If recovery or intervention was not evaluated, this is indicated as not assessed.

Model (Reference)	Species	Stress Paradigm/Duration	Major Reproductive Outcomes	Oxidative Stress/Mechanistic Endpoints	Notes (Intervention/Reversibility)
Chronic intermittent stress (restraint + swim) [43]	Rat	60 days; recovery assessed after cessation	Reduced sperm count and motility; increased abnormal morphology; germ cell loss/apoptosis; reduced testosterone	Increased testicular MDA; >50% decrease in SOD, CAT, and GPx activity	Reversibility: incomplete —many oxidative and sperm defects not fully normalized after a prolonged recovery period (reported as ~4 months)
Chronic restraint stress ± betaine [42]	Mouse	Chronic restraint stress (protocol-dependent)	Testicular damage and stress-related reproductive impairment (model of chronic stress)	Oxidative stress increased under stress; betaine attenuated oxidative and tissue injury	Intervention: betaine partially attenuated stress-related oxidative and tissue injury; Reversibility: after stress cessation not assessed
Chronic psychological stress (behavioral paradigm) [125]	Rat	Chronic psychological stress (duration per protocol)	Seminiferous tubule disruption; reduced sexual/reproductive behavior (female preference; mating attempts)	Increased testicular MDA and nitric oxide levels	Reversibility: not assessed (no post-stress recovery arm reported)
Forced swimming stress + vitamin C [126]	Rat	Forced swimming stress (experimental)	Stress-related decline in fertility-related endpoints	Redox-related injury implied; vitamin C improved outcomes	Intervention: vitamin C partially restored fertility-related endpoints in the stress model; Reversibility: after stress cessation not assessed
Chronic variable stress + vitamins C and E [127]	Rat	Chronic variable stress	Endocrine disruption (testosterone/cortisol) and stress-related reproductive impairment	Improved oxidative stress profile with vitamin C/E supplementation	Intervention: vitamins C and E improved endocrine and oxidative stress profile in the stress model; Reversibility: after stress cessation not assessed
Chronic unpredictable stress (BTB disruption) [128]	Mouse	Chronic unpredictable stress paradigm	Impaired sperm parameters in association with disrupted blood–testis barrier	Loss of tight junction/BTB integrity consistent with ROS-linked injury	Mechanistic barrier disruption model; Reversibility: after stress cessation or intervention not assessed
Chronic unpredictable stress (redox/inflammation signaling) [129]	Mouse	Chronic unpredictable stress exposure	Testicular dysfunction with altered germ cell junction dynamics	Modulation of Nrf2/HO-1 and IKbeta/NF-kappaB pathways consistent with pro-oxidant/pro-inflammatory shift	Mechanistic redox-sensitive signaling changes; Reversibility: not assessed
Restraint stress + melatonin [130]	Mouse	Restraint stress	Testicular cell apoptosis and functional impairment under stress	Reduced oxidative stress and apoptosis via NF-kappaB/iNOS and Nrf2/HO-1 signaling	Intervention: melatonin partially ameliorated oxidative stress and apoptosis; Reversibility: after stress cessation not assessed
Chronic stress and ER stress activation [131]	Mouse	Chronic stress model	Testis damage associated with cellular stress responses	Overactivation of PERK-mediated endoplasmic reticulum stress (interacts with oxidative stress pathways)	ER stress pathway activation model; Reversibility: not assessed
Paternal stress and sperm microRNA remodeling [132,133]	Mouse	Chronic paternal stress paradigms	Offspring phenotype changes (e.g., altered HPA-axis regulation/stress responsivity)	Altered sperm microRNA content; experimental microRNA delivery to zygotes recapitulated selected traits	Epigenetic and transgenerational model; Reversibility: not assessed
Chronic stress in fish with offspring effects [134]	Zebrafish	Chronic unpredictable stress (male)	Reduced sperm quality; molecular alterations in progeny	Sperm/offspring molecular signatures implicating small RNA and stress pathways	Transgenerational model beyond rodents; Reversibility: not assessed

Abbreviations: BTB, blood–testis barrier; CAT, catalase; ER, endoplasmic reticulum; GPx, glutathione peroxidase; iNOS, inducible nitric oxide synthase; MDA, malondialdehyde; NF-kappaB, nuclear factor kappaB; NO, nitric oxide; Nrf2, nuclear factor erythroid 2-related factor 2; HO-1, heme oxygenase-1; IKbeta, IκB kinase β; PERK, protein kinase RNA-like endoplasmic reticulum kinase; ROS, reactive oxygen species; SOD, superoxide dismutase.

## Data Availability

No new data were created or analyzed in this study. Data sharing is not applicable to this article.
